# Using machine learning to classify temporal lobe epilepsy based on diffusion MRI

**DOI:** 10.1002/brb3.801

**Published:** 2017-08-30

**Authors:** John Del Gaizo, Neda Mofrad, Jens H. Jensen, David Clark, Russell Glenn, Joseph Helpern, Leonardo Bonilha

**Affiliations:** ^1^ Department of Neurology Medical University of South Carolina Charleston SC USA; ^2^ Department of Radiology and Radiological Science Medical University of South Carolina Charleston SC USA; ^3^ Ralph H. Johnson VA Medical Center Charleston SC USA

**Keywords:** diffusion kurtosis imaging, epilepsy, machine learning, Magnetic Resonance Imaging (MRI), support vector machines

## Abstract

**Background:**

It is common for patients diagnosed with medial temporal lobe epilepsy (TLE) to have extrahippocampal damage. However, it is unclear whether microstructural extrahippocampal abnormalities are consistent enough to enable classification using diffusion MRI imaging. Therefore, we implemented a support vector machine (SVM)‐based method to predict TLE from three different imaging modalities: mean kurtosis (MK), mean diffusivity (MD), and fractional anisotropy (FA). While MD and FA can be calculated from traditional diffusion tensor imaging (DTI), MK requires diffusion kurtosis imaging (DKI).

**Methods:**

Thirty‐two TLE patients and 36 healthy controls underwent DKI imaging. To measure predictive capability, a fivefold cross‐validation (CV) was repeated for 1000 iterations. An ensemble of SVM models, each with a different regularization value, was trained with the subject images in the training set, and had performance assessed on the test set. The different regularization values were determined using a Bayesian‐based method.

**Results:**

Mean kurtosis achieved higher accuracy than both FA and MD on every iteration, and had far superior average accuracy: 0.82 (MK), 0.68 (FA), and 0.51 (MD). Finally, the MK voxels with the highest coefficients in the predictive models were distributed within the inferior medial aspect of the temporal lobes.

**Conclusion:**

These results corroborate our earlier publications which indicated that DKI shows more promise in identifying TLE‐associated pathological features than DTI. Also, the locations of the contributory MK voxels were in areas with high fiber crossing and complex fiber anatomy. These traits result in non‐Gaussian water diffusion, and hence render DTI less likely to detect abnormalities. If the location of consistent microstructural abnormalities can be better understood, then it may be possible in the future to identify the various phenotypes of TLE. This is important since treatment outcome varies dependent on type of TLE.

## 1 INTRODUCTION

The structural changes that may be unique to medial temporal lobe epilepsy (TLE) have been continually investigated to better understand the mechanisms associated with disease development and to define targets for treatment. Historically, TLE imaging findings have confirmed the existence of temporal and extratemporal areas of neuronal loss (Keller & Roberts, 2008); with more recent studies demonstrating that microstructural abnormalities are extensive and pervasively distributed (Bonilha et al., 2015). Since most structural brain studies have investigated group‐wise differences, it remains unclear whether microstructural abnormalities in TLE are consistently observed across all patients (and could therefore be used to classify TLE vs. individuals without TLE), or whether there is a high degree of variability beyond a common underlying pattern.

This question is important since the treatment outcomes of TLE are variable and not completely predictable based on clinical data (Engel et al., 2003; Spencer et al., 2005); indicating that there are different TLE phenotypes that remain unidentified, but could be discerned based on imaging, if the type and the location of regular TLE abnormalities were better understood. In this study, we tested the hypothesis that microstructural abnormalities are present within a common pattern in most subjects with TLE. We employed a machine‐learning algorithm to assess the accuracy with which individuals with TLE could be correctly classified as having epilepsy based on voxel‐based microstructural abnormalities detected by diffusion MRI, including Diffusional Kurtosis Imaging (DKI). DKI is a postprocessing approach for diffusion MRI with multiple b‐values that takes into account non‐Gaussian water molecule diffusion properties (Jensen et al., 2005). Since water diffusion in the brain is known to follow a non‐Gaussian pattern, DKI captures more structural information than DTI–based measures, which neglect diffusional non‐Gaussianity (Jensen et al., 2005). From DKI it is possible to calculate voxel‐wise mean kurtosis (MK), a measure of non‐Gaussianity, as well as traditional voxel‐wise diffusion tensor imaging parameters such as mean diffusivity (MD) and fractional anisotropy (FA).

We aimed to identify which extrahippocampal temporal lobe voxels were consistently abnormal in TLE, therefore serving as good disease classifiers. Moreover, we also tested which diffusion MRI measure (MK, MD, FA) was more consistently abnormal in each voxel, with the intent of providing anatomical and microstructural insight into TLE related abnormalities.

## 2 MATERIALS AND METHODS

### 2.1 Subjects

Thirty‐two TLE patients and 36 healthy controls were assessed in this study. This cohort was previously reported in a study that revealed DKI‐based, voxel‐based abnormalities in epilepsy (Bonilha et al., 2015). That study was not designed to assess individualized patterns of abnormalities or diffusion‐based classification accuracy, which is the novel purpose of this study. Only left‐sided TLE patients were used. As it is well known in the literature, left TLE is associated with a more widespread and homogeneous pattern of abnormalities compared with right TLE (Bonilha et al., 2007; Kemmotsu et al., 2011; Pustina et al., 2015; Ahmadi et al., 2009). Since the purpose of this study is to assess the classification algorithm, we opted to use a more regular cohort. All TLE patients met the International League Against Epilepsy criteria for diagnosis (Berg et al., 2010; Shorvon, 2011; Commission on Classification and Terminology of the International League Against Epilepsy, 1989). They were recruited from the Comprehensive Epilepsy center at the Medical University of South Carolina. The mean age of patients was 44.8 ± 16.7 years, with 22 females. This was a consecutive cohort of TLE patients, and not all patients were medication refractory. Their clinical characteristics can be appreciated in Table S1. The control population consisted of individuals with no neurological history or risk for epilepsy. Sex and age distribution differences between the patient and control groups were tested using a Chi‐squared and a *t*‐test, respectively, and were not statistically significant (*p* = 0.85 and *p* = 0.21).

### 2.2 Image acquisition

A 3T Magnetom Verio MRI scanner (Siemens, Erlangen, Germany) with a 12‐channel coil head was used to image all subjects. Diffusion imaging parameters were: a twice‐refocused echo‐planar imaging sequence with diffusion weightings of *b* = 0, 1,000, and 2,000 s/mm^2^, 30 diffusion‐encoding directions with number of excitations (NEX) = 1 (NEX = 10 for *b* = 0), repetition time = 8,500 ms, echo time = 98 ms, field‐of‐view = 222 × 222 mm^2^, a matrix size of 74 × 74, a parallel imaging factor of 2, 3 mm slice thickness, and 40 axial slices. No partial Fourier encoding was used. The imaging acquisition parameters are further specified in our previous work (Bonilha et al., 2015).

### 2.3 Image processing

Voxel‐based scalar diffusion measures were obtained using the software diffusional kurtosis estimator (DKE) (https://www.nitrc.org/projects/dke/). The probabilistic white matter map in MNI152 space distributed with software SPM 8 (http://www.fil.ion.ucl.ac.uk/spm/ software/spm8/) was nonlinearly transformed into native diffusion space and used for selection of white matter voxels as described below. The parameters MD and FA were estimated without using the *b* = 2,000 s/mm^2^ images in order to mimic a typical DTI analysis, while the MK calculation required the use of all the images for all the *b*‐values.

### 2.4 Statistical analyses with machine learning

We employed support vector machines (SVM) to analyze voxel contribution to epilepsy status. The diffusion measures compared were DKI‐derived MK, and DTI‐derived MD and FA. For each measure, the white matter voxels located in the left temporal lobe for the subjects were transformed into a matrix of size number of subjects by number of voxels. White matter voxels were those located in areas with 20% or greater probability of belonging to white matter in accordance with the probabilistic white matter map transformed into diffusion space. Left temporal lobe locations were identified using a mask derived from the Talairach Atlas in MNI space. Specifically, the atlas was transformed into diffusion space and a mask was created that corresponded to a union of all the ROIs associated with the left temporal lobe.

### 2.5 Prediction

Our classification pipeline consisted of training 19 different linear‐kernel SVM models from 19 different cost values (C) on the training set (D), and then performing a weighted average over the predictions of the 19 models for each subject in the test set (V) (Bishop, 2006) :

$$H=\left\{\left(\sum_{C}p_{C=c|D}*p_{1|C=c,D,i}\right)>\;0.5|i\;\in \;V\right\}$$

In the above equation, the weight of a model trained with a particular cost (C=c) is given by: $p_{C=c|D}$. These weights form a valid probability distribution as they sum to 1. Each classification model also outputs a probability, $p_{1|C=c,D,i}$, which is the probability the model assigns the i ^th^ test subject of being a patient. If the weighted average of the models’ outputs is greater than 0.5, then the subject is predicted to be a patient.

It is important to note that support vector machines do not directly output probabilities, but a score for each subject which ranges from −∞ to ∞. We employed a simple logistic link function to transform each score to a probability of being a patient ($p_{1|C=c,D,i}$). Further details are explained in the supplementary material.

A fivefold stratified cross‐validation scheme was executed, and the C vector was recalculated for the new D from each run of the cross‐validation. The folds were stratified to ensure a consistent patient to control ratio. To account for the variance associated with running fivefold cross‐validation on small datasets, we repeated this pipeline for 1,000 experimental iterations. Each iteration was unique from the other iterations in the random allocation of the data between the five folds.

The entire pipeline was written in Matlab (The MathWorks I, 2017), the implementation was placed online, and the URL can be found in the supplementary material.

### 2.6 Finding Values for the Cost Hyperparameter (*C*)

Our first step for generating the inputs for the weighted average was to calculate the vector of cost values (C). The mean of C was defined as the minimum cost value ($C_{\mu }$) required to fit D without any classification error on D. The methodology used to determine this minimum fit is described in the supplementary material. It is important to note that though a model fit with $C_{\mu }$ had no classification error on D, it still was not a maximum fit on D as the probability outputs from the model could be further optimized.

**Figure 1:**
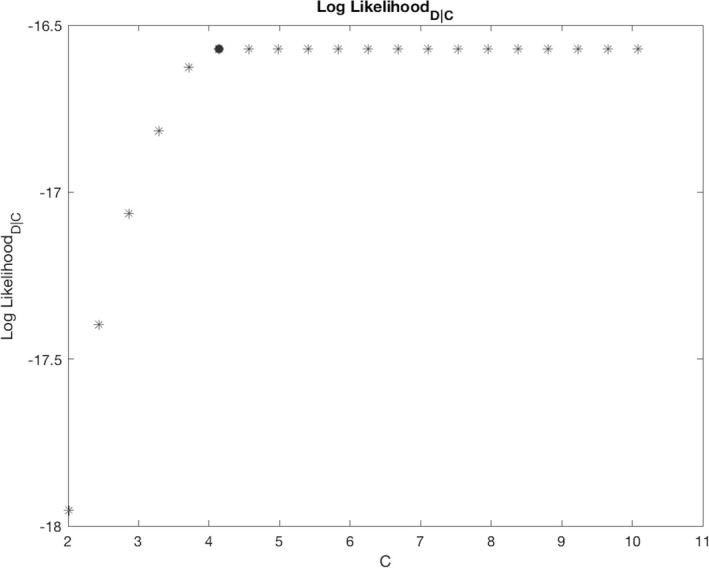
The fit on the training data ($LL_{D|C}$) increases approximately monotonically with C until a particular point, and then remains flat. The minimum (C=c) at which $LL_{D|C=c}=\max\left(LL_{D|C}\right)$ is depicted as a red dot and used as maximum value in the C vector

We then calculated the minimum cost required to have a maximum fit on D ($C_{\max}$). In other words, the probability outputs could not be further improved with increased C. The fit increases approximately monotonically with C until a particular value is reached, at which point the fit score remains flat. Slight noise deviations from the monotonic increase were smoothed with a length‐3 median filter. The fit was assessed with log‐likelihood ($LL_{D|C}$), the procedure for calculating the log‐likelihood for a given c ($LL_{D|C=c}$) is explained in the supplementary material. This optimization can be visualized in Figure 1.

From $C_{\max}$ and $C_{\mu }$, the C vector was found as:

$$\left\{C_{\mu }+\Delta *\frac{i}{9}|i\in -9\cdot \cdot \cdot 9\right\}$$

where $\Delta =C_{\max}-C_{\mu }$.

Calculating the model weights ($p_{C}|D$)

The model weights ($p_{C|D}$) were calculated through the Bayesian formula:

$$p_{C|D}=L_{D|C}*p_{C}/p_{D}$$

In the above formula, $L_{D|C}$ can be viewed as the evidence in Bayesian derivations, $p_{C}$ can be viewed as the prior, and dividing by $p_{D}$ scales the vector in the numerator ($L_{D|C}*p_{C}$) so that $p_{C|D}$ sums to 1 and hence is a proper probability distribution.

**Figure 2:**
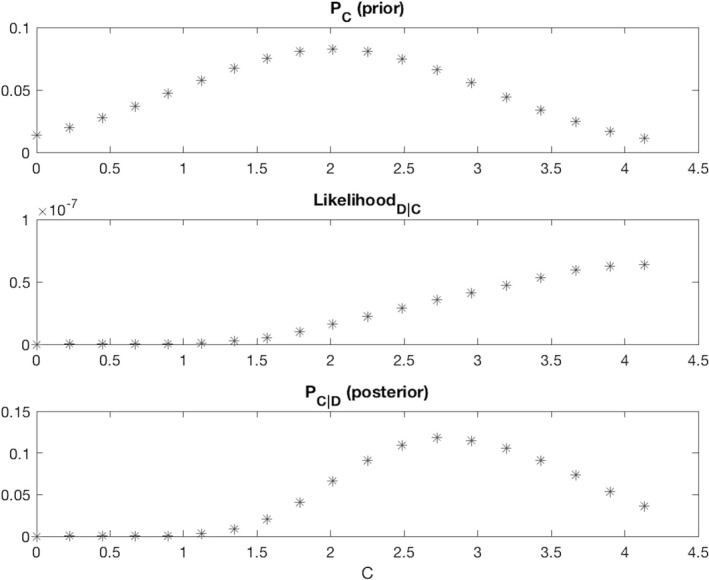
The weight assigned to each model is determined by the fit on the training data (second row) multiplied with a Gaussian prior that has a regularizing effect (first row). The final weight distribution (third row) reflects the influence from both distributions

The log of the evidence (the log‐likelihood, $LL_{D|C}$) was already calculated to find the C vector. $LL_{D|C}$ was converted to a likelihood ($L_{D|C}$) through the formula $\left\{e^{LL_{D|C=c}}|c\in C\right\}$, where each value indicates the likelihood of D given a model trained with C=c. An example $L_{D|C}$ can be seen in the middle panel of Figure 2.

Typically, $p_{C}$ is estimated from other experiments or data, but such information was not available. However, we did not desire “uniformed” flat priors because it was important to regularize $L_{D|C}$ (decrease the weights of the higher cost models) to prevent over‐fitting. Therefore, $p_{C}$ was defined as a Gaussian distribution with a mean at $C_{\mu }$, and a standard deviation of *C*_σ_ = Δ/2. The values were scaled to ensure that $p_{C}$ sums to 1. We selected $C_{\mu }$ as the point with maximal probability (the mean), because a model will almost always have higher generalization error than training error, therefore the model needs to at least fit D. However, such sparse datasets are highly susceptible to over‐fitting, therefore we chose a distribution with small weights for models that strongly fit D: for example, since, its corresponding weight will approximate 0.0275. An example of $p_{C}$ can be found in the top panel of Figure 2.

As both $L_{D|C}$ and $p_{C}$ were represented as vectors of size (19 by 1), the scalar $p_{D}$ was simply found through a dot product: $p_{D}={L_{D|C}}^{\prime }\cdot p_{C}$.

The final $p_{C|D}$ derived from the $p_{C}$ and $L_{D|C}$ examples used in this explanation can be seen in the bottom panel of Figure 2.

### 2.7 Generating coefficients

On each run of the cross‐fold validation, a $\beta_{C=c}$ vector of coefficients was generated for each of the 19 SVM models from the training data (D) associated with the current run (R=r). The vectors were aggregated into a single vector per run with a weighted average: $\beta_{R=r}=\sum_{C}p_{C=c|D}*\beta_{C=c}$. The coefficients per run were averaged across runs to generate a vector for each of the 1,000 experimental iterations: $\beta_{I=i}=\frac{1}{5}\sum_{R}\beta_{R=r}$. Finally, the $\beta_{I}$ vectors were aggregated into four vectors as shown below:

- Mean vector
  - $\beta_{\mu }=\frac{1}{1000}\sum_{i=1}^{1000}\beta_{I=i}$
- Standard deviation vector
  - $\beta_{\sigma }=\surd \left(\frac{1}{1000}\sum_{i=1}^{1000}{\left(\beta_{I=i}-\beta_{\mu }\right)}^{2}\right)$
- Positive run count vector
  - $\beta_{+}=\sum_{i=1}^{1000}\beta_{I=i}>0$
- Negative run count vector
  - $\beta_{-}=\sum_{i=1}^{1000}\beta_{I=i}<0$

Since the SVMs used linear kernels, these values could be directly interpreted. Voxels with negative coefficients had higher intensities for controls compared to patients, and positive coefficients corresponded to lower intensities for controls compared to patients.

### 2.8 Ethical publication statement

We confirm that we have read the Journal's position on issues involved in ethical publication and affirm that this report is consistent with those guidelines.

## 3 RESULTS

### 3.1 Individual measures

**Figure 3:**
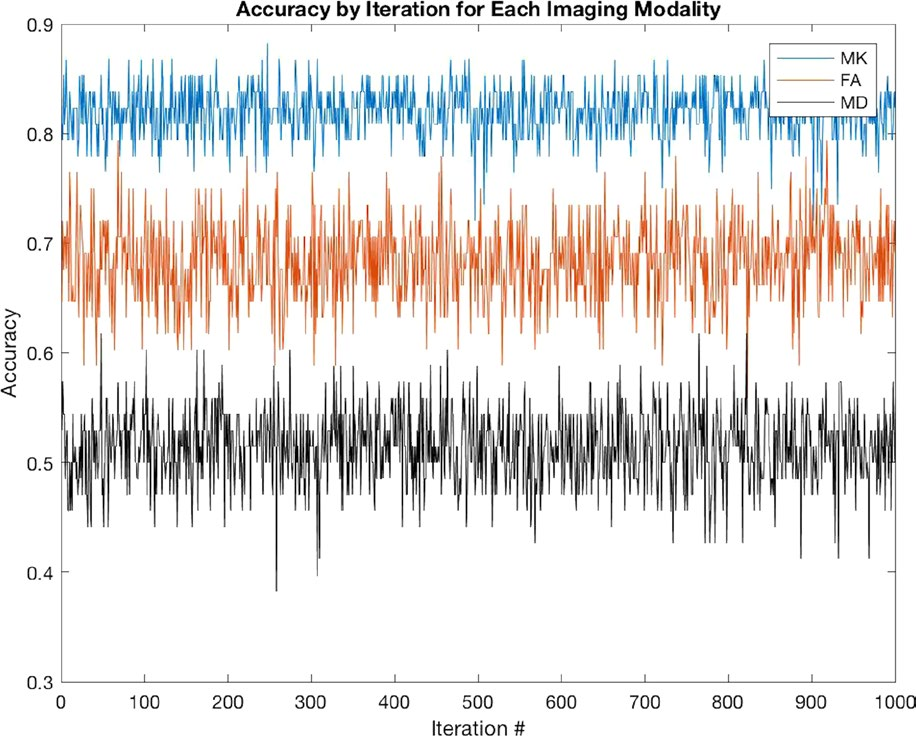
At each iteration of the experiment, the subjects are randomly allocated among five folds. These subjects are used to train and test the models derived from the different measures. MK has higher accuracy than both FA and MD on all 1,000 iterations of the experiment

**Figure 4:**
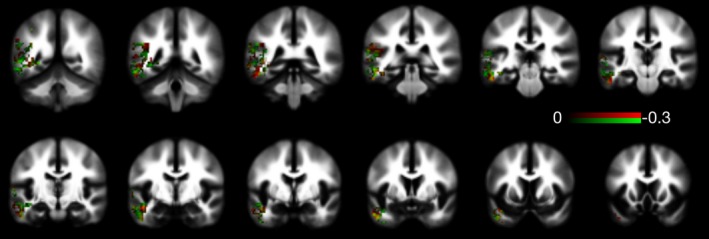
This mosaic demonstrates which FA and MK voxels most contributed to the classification model. Voxels colored in red were those in which lower values of MK had higher weight toward classifying individuals as belonging to the group of patients. Similarly, the voxels colored in green were those in which lower FA values contributed toward classifying the individuals as patients. The color bar represents the weights, whereas lower negative weights indicated a higher influence in the more towards classification as patients. Finally, voxels colored in yellow (red + green) where those where both the FA and MK values contributed to the classification

As shown in Table 1, the classifier trained and tested with MK best classified TLE, followed by FA and then MD. As shown in Figure 3 and Table 1, MK scored higher prediction metrics for accuracy, F1‐score, sensitivity, and specificity. With regard to accuracy and F1‐score, MK out‐performed the other measures for all 1,000 iterations. Figure 4 depicts the most contributory voxels for MK and FA.

**Table 1:** Predictive values following SVM vector analysis by diffusion measure

Measure	Accuracy	F1 Score	Sensitivity	Specificity
MK	0.820 ± 0.023	0.800 ± 0.026	0.765 ± 0.032	0.870 ± 0.031
FA	0.683 ± 0.037	0.642 ± 0.047	0.606 ± 0.058	0.752 ± 0.041
MD	0.514 ± 0.035	0.400 ± 0.047	0.345 ± 0.049	0.664 ± 0.049

FA also clearly out‐performed MD. However, the accuracy associated with FA was not significantly higher than what can be achieved with Gaussian noise given a small dataset (Combrisson & Jerbi, 2015).

**Figure 5:**
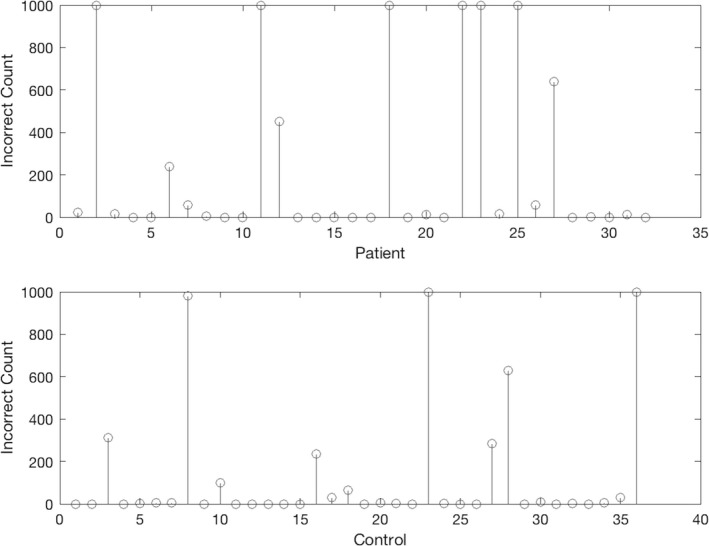
This plot depicts the number of times each subject was misclassified after 1,000 experimental iterations. There were six patients and three controls that the models did not correctly classify for any of the iterations

As shown in Figure 5, certain subjects were far more likely to be misclassified. In particular, there were six patients and three controls that MK did not correctly predict in any of the 1,000 iterations.

## 4 DISCUSSION

MK‐derived classifiers were far more accurate for classifying TLE than FA‐ or MD‐derived classifiers. However, certain subjects were always misclassified regardless of the data allocation across the folds.

Further insight into the relative contribution of each voxel location can be gained by assessing the anatomical distribution of abnormalities that contribute to classification. As seen in Figure 4, the implicated voxels in MK were distributed within the inferior medial aspect of the temporal lobes, which may represent areas of higher fiber crossing, more complex fiber anatomy, or more axonal changes associated with epilepsy. Therefore, it appears these measures may reflect components of microstructural pathology that are subtly different, but important in the classification of TLE.

Our results corroborate previous studies that have highlighted the sensitivity of DKI as compared to standard DTI when measuring microstructural abnormalities in adult and pediatric patients with TLE (Bonilha et al., 2015; Gao et al., 2012). The microstructural complexity and compartmentalization of brain tissue result in non‐Gaussian water diffusion, which DTI cannot detect. By quantifying this diffusional non‐Gaussianity with measures such as MK, DKI more fully reveals micropathologic changes that may be associated with inflammation and cell loss (Winston, 2015).

Our results achieve comparable performance metrics to previous literature that applies SVM to neuroimaging data to classify TLE. An SVM study using T1‐weighted images performed by Rudie et al. achieved a prediction accuracy of up to 81% for patients with TLE compared to those with other structural abnormalities, and it additionally found correlations between predictive value and clinical disease progression (Rudie, Colby, & Salamon, 2015).

Furthermore, our results indicate that microstructural abnormalities in TLE (here exemplified by left TLE) may enable an accurate classification with kurtosis‐based imaging. This is important since it demonstrates that a pattern of microstructural pathology is common across individuals with TLE and therefore forms a structural mainstay for the disease. Importantly, this approach is not intended only to diagnose epilepsy, but may be able to identify a common pathological pattern that could be used as a decision support tool for clinical assessments in the future. We recommend that future analyses investigate the subjects that were consistently misclassified. It is possible that these subjects contain subtle but important features that distinguish patients from controls, which may be overshadowed by more general differences between the groups.

## CONFLICT OF INTEREST

None of the authors have any conflict of interest related to this manuscript to disclose.
