# Supplementary material for: Using machine learning to classify temporal lobe epilepsy based on diffusion MRI
Source: Brain Behav. 2017 Aug 30;7(10):e00801. doi: 10.1002/brb3.801 (PMC5651385; doi:10.1002/brb3.801)
Supplement: Supplementary file 1 [file BRB3-7-e00801-s001.docx]

| Patient Number | Age (years) | Sex | Age of Epilepsy Onset (years) | Frequency (6 months) | MRI Results |
| --- | --- | --- | --- | --- | --- |
| 1 | 20 | F | 19 | 1 | Left hippocampal atrophy |
| 2 | 45 | M | 27 | 2 | Left hippocampal atrophy |
| 3 | 40 | M | 37 | .2 | Left hippocampal atrophy |
| 4 | 34 | M | 15 | 1 | Normal |
| 5 | 18 | F | 3 | 72 | Left hippocampal atrophy |
| 6 | 20 | M | 19 | .2 | Left hippocampal atrophy |
| 7 | 45 | F | 33 | 2 | Left hippocampal atrophy |
| 8 | 63 | F | 57 | 1 | Normal |
| 9 | 57 | F | 50 | 6 | Left hippocampal atrophy |
| 10 | 37 | F | 28 | 2 | Left hippocampal atrophy |
| 11 | 23 | F | 17 | 6 | Left hippocampal atrophy |
| 12 | 21 | F | 20 | 1 | Left hippocampal atrophy |
| 13 | 18 | F | 5 | 3 | Left hippocampal atrophy |
| 14 | 59 | F | 42 | 3 | Left hippocampal atrophy |
| 15 | 22 | M | 10 | .5 | Left hippocampal atrophy |
| 16 | 58 | F | 55 | 1 | Left hippocampal atrophy |
| 17 | 57 | F | 10 | 2 | Left hippocampal atrophy |
| 18 | 67 | F | 66 | 6 | Normal |
| 19 | 46 | M | 3 | 12 | Left hippocampal atrophy |
| 20 | 51 | F | 50 | 12 | Normal |
| 21 | 57 | F | 35 | 24 | Left hippocampal atrophy |
| 22 | 57 | F | 2 | 6 | Left hippocampal atrophy |
| 23 | 56 | M | 30 | 6 | Left hippocampal atrophy |
| 24 | 46 | F | 35 | .5 | Left hippocampal atrophy |
| 25 | 27 | F | 27 | .2 | Left hippocampal atrophy |
| 26 | 76 | F | 30 | 6 | Left hippocampal atrophy |
| 27 | 43 | M | 5 | 3 | Left hippocampal atrophy |
| 28 | 57 | F | 52 | 3 | Normal |
| 29 | 65 | M | 59 | 1 | Left hippocampal atrophy |
| 30 | 36 | M | 17 | 1 | Left hippocampal atrophy |
| 31 | 62 | F | 62 | .2 | Left hippocampal atrophy |
| 32 | 37 | F | 33 | 6 | Left hippocampal atrophy |
