# Supplementary material for: Using machine learning to classify temporal lobe epilepsy based on diffusion MRI
Source: Brain Behav. 2017 Aug 30;7(10):e00801. doi: 10.1002/brb3.801 (PMC5651385; doi:10.1002/brb3.801)
Supplement: Supplementary file 2 [file BRB3-7-e00801-s002.docx]

**Using machine learning to classify Temporal Lobe Epilepsy based on diffusion MRI**

**Converting an SVM output to a probability**

The derivation of $p_{1|C=c, D, i}$ is described as follows:

1. For a given $c$, train a linear SVM model on $D$ to obtain $\beta_{C=c, D}$ coefficients and a bias ($b_{C=c, D}$).
2. For each subject, use $\beta_{C=c, D}$ and $b_{C=c, D}$ to calculate a score.
   - $s\left( i \right)= \beta_{C=c,D}x^{i}+b_{C=c,D}$, where $x^{i}$ is the white matter vector for subject $i$
3. Apply the sigmoid function to convert the score from 2, which ranges from $-\infty$ to $\infty$, to a probability of being a patient.

$p_{1|C=c,D,i}= 1/{(1+e^{-s(i)})}$

**Finding the minimum cost ($C_{\mu}$) to fit $D$ with no classification error**

1. Calculate the probability that the model trained with $c$ predicts the $i$^th^ subject in $D$ as a patient ($p_{1|C=c, D, i}$), as described previously.
2. Predict if subject $i$ is a patient or control.

$$h^{i}=p_{1|C=c,D,i}> .5$$

3. Apply steps 1-2 to all subjects in $D$ to obtain a prediction accuracy
4. Iterate over different values of $c$ using Newton-based optimization. On each iteration, apply steps 1 – 3 to find the minimum $c$ with no prediction error on $D$
   $argmin C, subject to: \forall i \in D: h^{i}=y^{i}$
   - - where $y^{i}$ indicates the subject’s true value.

**Calculating the log-likelihood for a given** $c$(${LL}_{D|C=c}$)

1. Calculate the probability of a correct prediction for each subject in $D$.

$$p_{D|C=c,i}=y^{i}*p_{1|C=c,D,i}+(1-y^{i})(1-p_{1|C=c,D,i})$$

2. Calculate log-likelihood of the joint probability

$${LL}_{D|C=c}= log(\prod_{i=1}^{D} p_{D|C=c,i})=\sum_{i=1}^{D} log(p_{D|C=c,i})$$

**Machine Learning Implementation**

The machine learning code was written in Matlab and requires the Statistics and Machine Learning Toolbox. We have placed the code on Github, which is free for the scientific community to download and reproduce the methods. A readme file is included on Github to explain how to use. It can be accessed from the link below:

https://github.com/JDMusc/epi_prediction_ml.git
